# ACL reconstruction combined with anterolateral structures reconstruction for treating ACL rupture and knee injuries: a finite element analysis

**DOI:** 10.3389/fbioe.2024.1437684

**Published:** 2024-08-06

**Authors:** Huizhi Wang, Gai Yao, Kaixin He, Zimin Wang, Cheng-Kung Cheng

**Affiliations:** ^1^ School of Biomedical Engineering and Engineering Research Center for Digital Medicine of the Ministry of Education, Shanghai Jiao Tong University, Shanghai, China; ^2^ Center for Intelligent Medical Equipment and Devices (iMED), University of Science and Technology of China, Suzhou, Jiangsu, China; ^3^ The Fifth Medial Center of Chinese PLA General Hospital, Beijing, China; ^4^ Department of Orthopedic Surgery, Shanghai Ninth People’s Hospital, Shanghai Jiao Tong University School of Medicine, Shanghai, China

**Keywords:** anterior cruciate ligament reconstruction, anterolateral structures reconstruction, pivot shift, articular stress, combined injury

## Abstract

**Introduction:** The biomechanical indication for combining anterolateral structures reconstruction (ASLR) with ACL reconstruction (ACLR) to reduce pivot shift in the knee remains unclear. This study aims to investigate knee functionality after ACL rupture with different combinations of injuries, and to compare the effectiveness of ALSR with ACLR for treating these injuries.

**Methods:** A validated finite element model of a human cadaveric knee was used to simulate pivot shift tests on the joint in different states, including 1) an intact knee; 2) after isolated ACL rupture; 3) after ACL rupture combined with different knee injuries or defect, including a posterior tibial slope (PTS) of 20°, an injury to the anterolateral structures (ALS) and an injury to the posterior meniscotibial ligament of the lateral meniscus (LP); 4) after treating the different injuries using isolated ACLR; v. after treating the different injuries using ACLR with ALSR. The knee kinematics, maximum von Mises stress (Max.S) on the tibial articular cartilage (TC) and force in the ACL graft were compared among the different simulation groups.

**Results and discussion:** Comparing with isolated ACL rupture, combined injury to the ALS caused the largest knee laxity, when a combined PTS of 20° induced the largest Max.S on the TC. The joint stability and Max.S on the TC in the knee with an isolated ACL rupture or a combined rupture of ACL and LP were restored to the intact level after being treated with isolated ACLR. The knee biomechanics after a combined rupture of ACL and ALS were restored to the intact level only when being treated with a combination of ACLR and ALSR using a large graft diameter (6 mm) for ALSR. However, for the knee after ACL rupture combined with a PTS of 20°, the ATT and Max.S on the TC were still greater than the intact knee even after being treated with a combination of ACLR and ALSR. The finite element analysis showed that ACLR should include ALSR when treating ACL ruptures accompanied by ALS rupture. However, pivot shift in knees with a PTS of 20° was not eliminated even after a combined ACLR and ALSR.

## 1 Introduction

Rupture of the anterior cruciate ligament (ACL) can lead to knee laxity and often requires reconstruction surgery to stabilize the joint (Musahl et al., 2022). However, a positive pivot shift (excessive tibial translations and rotations) has been commonly reported after ACL reconstruction (ACLR) (Guenther et al., 2015), with an incidence rate of up to 25% (Sonnery-Cottet et al., 2017). The positive pivot shift indicates that the ACL graft is not bearing the expected load, which could lead to abnormal loads on other joint tissues (Du et al., 2016; Wang et al., 2020b), such as abnormal stress on the cartilage that may gradually develop into long-term osteoarthritis (Barenius et al., 2014; Shen et al., 2021).

Studies have found that people with concomitant knee injuries or anatomical defects experience greater pivot shifts. These concomitant injuries include ruptures of the anterolateral structures (ALSs), including the anterolateral ligament (ALL) and anterolateral capsule (ALC) (Song et al., 2017), rupture of the posterior meniscotibial ligament of the lateral meniscus (LP) (Tang et al., 2019; Ni et al., 2022), or instances of greater posterior tibial slope (PTS) (Ansari et al., 2017). However, few studies have explored the differences in the severity of pivot shifts among patients with various injuries and defects, and the surgical strategies that should be used to treat patients with such injury patterns remain unclear.

Anterolateral structures reconstruction (ALSR) (Chen et al., 2021), also termed as anterolateral ligament reconstruction (ALLR), has been reported as an efficient method of reducing pivot shift of the knee after ACLR (Nitri et al., 2016; Sonnery-Cottet et al., 2017; Wood et al., 2018; Ueki et al., 2019). Through a cadaveric study, Nitri et al. (2016) found that anatomical ACLR combined with ALSR significantly improved the rotatory stability of the knee compared to isolated ACLR in the presence of concurrent ALL deficiency with ACL rupture. Ueki et al. (2019) found that using additional ALS augmentation in patients with high preoperative pivot shift reduced the pivot-shift acceleration when compared to isolated ACLR. To provide a comprehensive reference for the anatomical and biomechanical basis of ALSR, an expert group consensus was published in 2017, containing a summary of the current scientific evidence and recommendations for improving surgical techniques (Sonnery-Cottet et al., 2017). The consensus document identified the importance of ALSR for people at high risk of pivot shifts, but the exact surgical indications remain unclear.

In the above context, the present study aimed to 1) explore differences in pivot shift (anterior and rotational displacements of the knee) and maximum stress (Max.S) values on the tibial cartilage (TC) when the knee was subjected to different combinations of injuries or defects accompanied by a ruptured ACL; further, the study 2) compares the effectiveness of using ALSR with ACLR for treating different combinations of injuries. Finite element analysis was used in this study, through which the variate was strictly controlled to one factor while maintaining the other factors constant. This permits the basic biomechanics of the joint to be examined clearly (Wang et al., 2022a). It was hypothesized that there would be considerable differences in the pivot shifts and articular stresses among knees with different injuries; further, by combining ACLR and ALSR, postoperative joint biomechanics can be restored in patients with certain injury patterns, while other injury patterns cannot achieve joint function restoration through ACLR and ALSR.

## 2 Materials and methods

A validated finite element model of a male human cadaveric knee joint (Wang et al., 2020b) was used in the current study to simulate pivot shift tests for different states of injury and repair: intact knee; isolated ACL rupture; ACL rupture with different combinations of knee injuries or defects, including rupture of the LP, ALS, and a PTS of 20°; ACLR using anatomical single-bundle grafts of various diameters; combined ALSR with ACLR. The knee kinematics, Max.S on the TC, and force in the ACL graft were compared among the different simulation groups to explore the differences in pivot shifts after different knee injuries as well as the effectiveness of ALSR combined with ACLR for restoring knee biomechanics when used to treat different knee injuries.

### 2.1 Development and validation of the finite element model of an intact cadaveric knee

**FIGURE 1:**
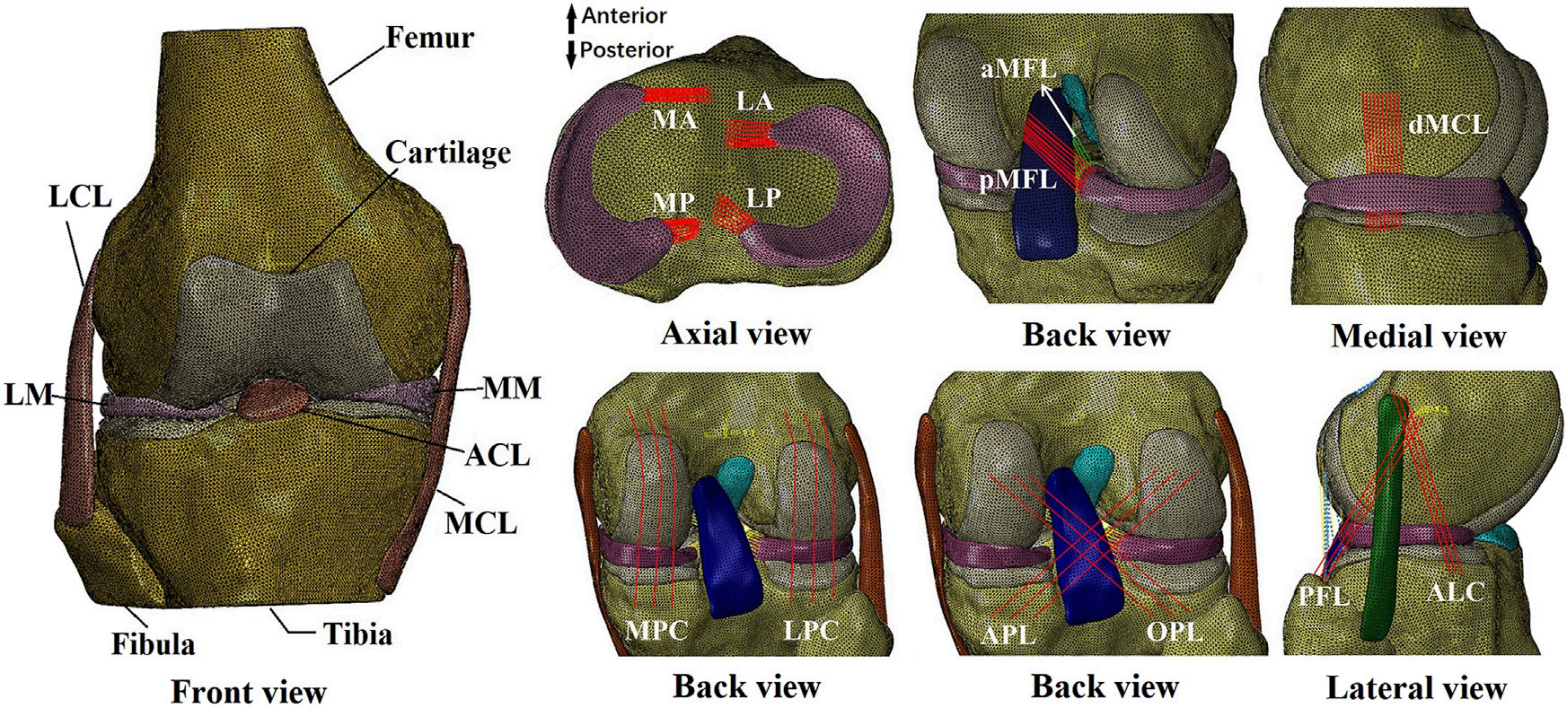
Finite element model of a human cadaveric right knee. LM, lateral meniscus; LCL, lateral collateral ligament; MM, medial meniscus; ACL, anterior cruciate ligament; MCL, medial collateral ligament; MA, anterior medial meniscotibial ligament; MP, posterior meniscotibial ligament; LA, anterior lateral meniscotibial ligament; LP, posterior lateral meniscotibial ligament; aMFL, anterior meniscofemoral ligament; pMFL, posterior meniscofemoral ligament; dMCL, deep medial collateral ligament; MPC, medial posterior capsule; LPC, lateral posterior capsule; APL, arcuate popliteal ligament; OPL, oblique popliteal ligament; PFL, popliteofibular ligament; ALC, anterolateral capsule.

The finite element model was built from a cadaveric human (45 years, male, 70 kg) knee joint (right side) (Figure 1) with prior approval from the Committee for Oversight of Research and Clinical Training Involving Decedents (Guenther et al., 2015). The knee was examined by an experienced orthopedic surgeon and determined to have normal tissue morphology with no observable injury or history of operation. The geometries of the knee structures were segmented using Mimics (Materialise N.V., Leuven, Belgium) from magnetic resonance images acquired with a slice thickness of 0.2 mm and scan resolution of 0.2 mm × 0.2 mm (field of view: 8 cm × 10 cm × 12 cm, TR = 53 ms, TE = 26.3 ms, field strength: 3.0 T). The model included bones (tibia, femur, and fibula), articular cartilage, meniscus, four major ligaments (posterior cruciate ligament, ACL, lateral collateral ligament, and medial collateral ligament), meniscal ligaments (anterior and posterior meniscotibial ligaments and meniscofemoral ligaments), capsule deep medial collateral ligament for medial stability, popliteofibular ligament with ALL and ALC for lateral stability, and four structures for maintaining posterior stability (lateral posterior capsule, medial posterior capsule, arcuate popliteal ligament, and oblique popliteal ligament).

The 3D model was meshed using 4-node tetrahedral elements in HyperMesh (Altair Engineering, Japan). Convergence tests were conducted to obtain optimized element sizes (loading condition: 2.5 mm tibial load with the femur totally fixed at full knee extension; output: force in the ACL). The optimized element had a side length of 1 mm, resulting in a total of 659,251 elements in the final intact knee model.

The material properties of the joint tissues were defined according to previous literature (Gupte et al., 2002; Song et al., 2004; Robinson et al., 2005; Hauch et al., 2010; Baldwin et al., 2012; Beidokhti et al., 2018; Wang et al., 2019) using Abaqus/CAE 2019 (Simulia, Inc., United States). The bones and cartilage were assumed to be linear isotropic elastic tissues (Young’s modulus = 0.4 GPa and 5 MPa; Poisson’s ratio = 0.33 and 0.46, respectively). The meniscus was assumed to be orthotropic elastic ( $E_{\theta }$ = 125 MPa, $E_{Z}$ = $E_{R}$ = 27.5 MPa, $G_{\theta R}$ = $G_{\theta Z}$ = 2 MPa, $G_{RZ}$ = 10.34, $V_{RZ}$ = 0.33, and $V_{\theta R}$ = $V_{\theta Z}$ = 0.1). The ligaments and knee capsules were defined as isotropic linear elastic tissues. The material coefficients for these tissues are shown in Table 1. Frictionless sliding was defined at the contact interfaces between the cartilages and menisci to permit sliding of the contact surfaces without penetration. Tie contacts were used to connect the ligaments/capsules to their bone interfaces and between the articular cartilage and corresponding bone surface so that there was no relative movements between the connected surfaces.

**TABLE 1:** Material coefficients for the ligaments and joint capsules obtained from literature (Gupte et al., 2002; Song et al., 2004; Robinson et al., 2005; Hauch et al., 2010; Baldwin et al., 2012; Beidokhti et al., 2018; Wang et al., 2022a).

Tissue	Stiffness (N/mm)	Young’s modulus (MPa)	Poisson’s ratio
ACL	--	168	0.4
PCL	258	--	--
MCL	--	179	0.4
LCL	--	224	0.4
MA	169	--	--
LA	216	--	--
MP	207	--	--
LP	130	--	--
aMFL	200	--	--
pMFL	206	--	--
dMCL	42	--	--
ALL	16	--	--
ALC	25	--	--
PFL	38	--	--
MPC	15	--	--

ACL, anterior cruciate ligament; PCL, posterior cruciate ligament; MCL, medial collateral ligament; LCL, lateral collateral ligament; MA, anterior meniscotibial ligament of the medial meniscus; LA, anterior meniscotibial ligament of the lateral meniscus; MP, posterior meniscotibial ligament of the medial meniscus; LP, posterior meniscotibial ligament of the lateral meniscus; aMFL, anterior meniscofemoral ligament; pMFL, posterior meniscofemoral ligament; dMCL, deep medial collateral ligament; ALL, anterolateral ligament; ALC, anterolateral capsule; PFL, popliteofibular ligament; MPC, medial posterior capsule; LPC, lateral posterior capsule; APL, arcuate popliteal ligament; OPL, oblique popliteal ligament.

The model was validated by comparing the calculated data with experimental data from previous studies (Kanamori et al., 2000; Wang et al., 2020c). The compared data included knee kinematics and force in the ACL under the following three loading conditions at a knee flexion angle of 30°, with the femur completely fixed and having six degrees of freedom and the tibia subjected to (a) an anterior load of 134 N, (b) an internal moment of 10 Nm, and (c) an internal moment of 10 Nm and a valgus moment of 10 Nm. The results in Table 2 show that the model calculations were all within the range of values reported in cadaveric experiments.

**TABLE 2:** Anterior tibial translation, internal tibial rotation, and *in situ* force in the ACL obtained from previous literature (Kanamori et al., 2000; Wang et al., 2019) and the current finite element model calculation under the following loading conditions applied at a joint flexion angle of 30°: (a) 134 N anterior tibial load, (b) 10 Nm internal tibial moment, and (c) 10 Nm internal tibial moment with 10 Nm valgus tibial moment.

	134 N anterior tibial load		10 Nm internal tibial moment		10 Nm valgus tibial moment + 10 Nm internal tibial moment	
	Anterior tibial translation (mm)	*In situ* force in the ACL (N)	Internal tibial rotation (°)	*In situ* force in the ACL (N)	Internal tibial rotation (°)	*In situ* force in the ACL (N)
Experimental [18, 41]	5.1	124	20.5 ± 3.5	42 ± 22	22.3 ± 3.5	69 ± 32
Computational (current study)	5.2	126	17.6	37	19.1	56

### 2.2 Simulation of isolated ACL rupture and ACL ruptures with various injuries or knee defects

**FIGURE 2:**
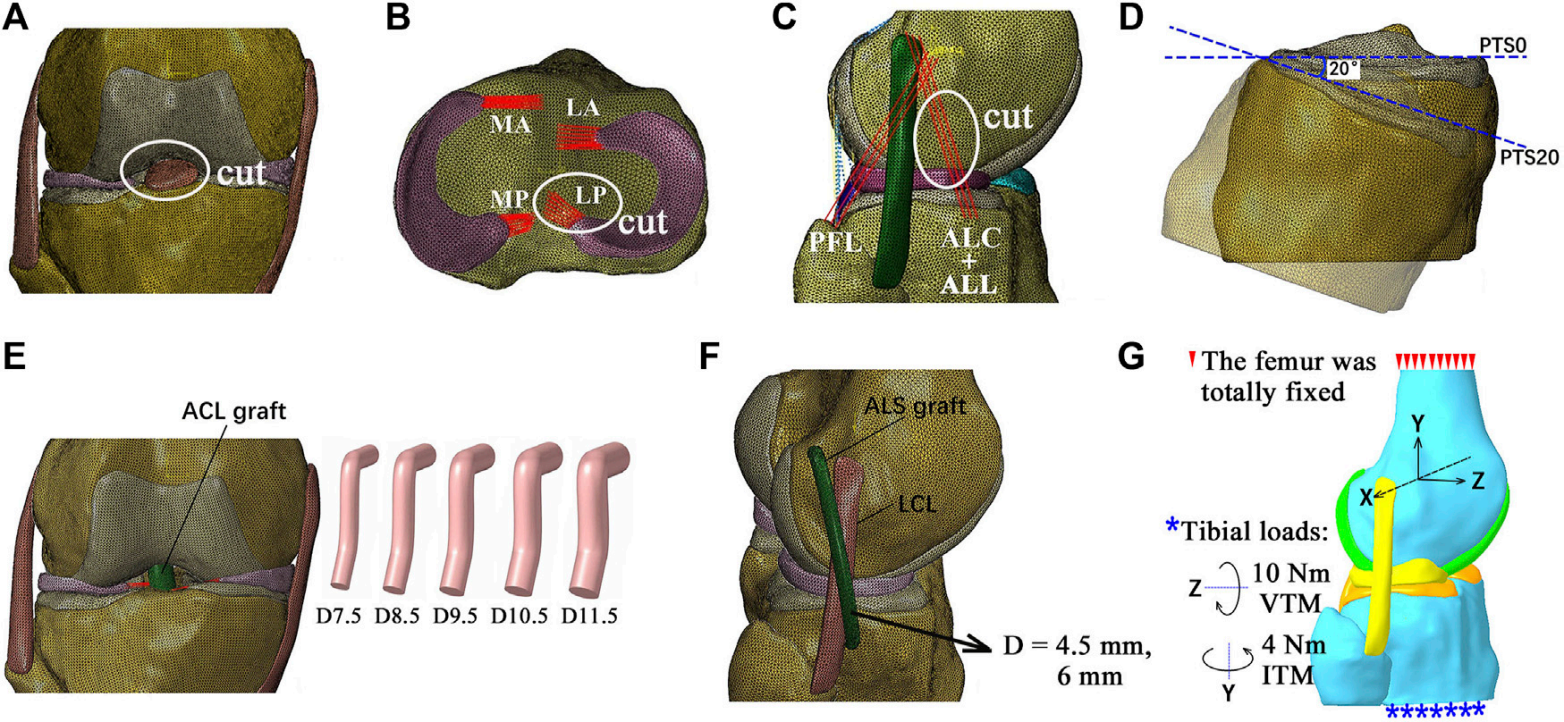
Diagrams of the finite element models simulating different combinations of knee injuries. **(A)** Simulation of an isolated ACL rupture by cutting the ACL. **(B)** Simulation of ACL rupture combined with rupture of the posterior lateral meniscotibial ligament (LP). **(C)** Simulation of ACL rupture combined with rupture of the anterolateral structures (ALSs), including the anterolateral ligament (ALL) and anterolateral capsule (ALC). **(D)** Simulation of a posterior tibial slope (PTS) of 20°. **(E)** Simulation of anatomical single-bundle (SB) ACL reconstructions (ACLRs) with different graft diameters. **(F)** ACLR combined with ALS reconstruction (ALSR) with ALS graft diameters of 4.5 mm and 6 mm. **(G)** Boundary and loading conditions for simulating pivot shifts. MA, anterior medial meniscotibial ligament; MP, posterior meniscotibial ligament; LA, anterior lateral meniscotibial ligament; PFL, popliteofibular ligament; D7.5, graft diameter of 7.5 mm; ITM, internal tibial moment; VTM, valgus tibial moment.

To simulate ruptured ACLs in intact and injured knees, the related tissues were removed from the model (Figure 2). To simulate a PTS, the tibial plateau was rotated around the medial–lateral axis of the joint in the sagittal plane while all other degrees of freedom were unconstrained, as depicted in a previous study (Voos et al., 2012) (Figure 2D). Akoto et al. (2020) showed that patients with ACLR failure and high-grade anterior knee laxity had PTSs ranging from 13° to 20°. In this study, a PTS of 20° was simulated to represent a severe case. The following injury/defect modes were simulated: isolated ACL rupture (Figure 2A); combined ruptures of the ACL and LP (Figure 2B); combined ruptures of the ACL and ALS (Figure 2C); ACL rupture with a PTS of 20° (Figure 2D).

### 2.3 Simulation of anatomical single-bundle ACLR with varying graft diameters

The anatomical single-bundle ACLR for treating the abovementioned combinations of knee injuries and defects was simulated (Figure 2E). The entrances for the bone tunnels were placed at the centers of the anatomical insertion sites. The angles of the femoral tunnel with the sagittal and axial planes were 25° and 45°, and those of the tibial tunnel were 25° and 65°, respectively (Wang et al., 2022b). The ACL graft was simulated as a cylindrical structure using Creo Parametric 8.0 (PTC, MA, United States), with its Young’s modulus set as 168 MPa (Wilson et al., 1999). The graft was simulated to be fixed to the bone tunnels by an endoscrew, which was also modeled as a cylinder (length of 10 mm and same diameter as the graft) (Wang et al., 2020a). The endoscrew was affixed to the graft at one end and in contact with the tunnel wall at its exterior surface so that there were no relative motions between these contact surfaces. The Young’s modulus and Poisson’s ratio of the endoscrew were respectively set to 110 GPa and 0.35 to simulate titanium material. The ACLR was simulated with different graft diameters (7.5, 8.5, 9.5, 10.5, and 11.5 mm) to determine the dimensions that were best able to restore knee functionality. The simulated bone tunnel diameters were similar to those of the graft.

### 2.4 Simulation of combined ALSR with ACLR

To evaluate the effectiveness of ALSR in restoring knee kinematics after injury to the ACL, the knee model was simulated with an isolated ACL rupture and treated using ALSR combined with ACLR (Figure 2F) and compared with the outcome after treatment through isolated ACLR (graft diameters: 8.5 mm and 10.5 mm). The diameter of the ALS graft was increased from 4.5 mm to 6 mm to explore the effect of the ALS graft diameter on the surgical outcome. Then, the effectiveness of combined ACLR and ALSR was evaluated for treating different combinations of knee injuries.

The surgical techniques recommended by the 2017 expert group consensus (Sonnery-Cottet et al., 2017) were used to simulate the ALSR in this study. Specifically, the femoral tunnel was placed 8 mm proximal and 4 mm posterior to the lateral epicondyle, and the tibial tunnel was placed 10 mm below the joint line at the halfway point between the center of the fibula head and Gerdy’s tubercle. A graft diameter of 4.5 mm was used, as suggested by the consensus, and the Young’s modulus was set as 618.4 MPa (Hamner et al., 1999). The Poisson’s ratio was the same as that of the other ligaments (0.4) in the model. The tunnel axes both lay in the sagittal plane. The angle of the femoral tunnel with respect to the transverse plane was 20°, and the angle of the tibial tunnel with respect to the transverse plane was 30°. The graft fixation method previously detailed for the ACLR model was also used for the ALSR models.

### 2.5 Loading conditions and outputs

To simulate pivot shifts, all models were loaded with an internal tibial moment of 4 Nm and a valgus tibial moment of 10 Nm at full knee extension (Wan et al., 2017; McLeod and Barber, 2023) (Figure 2G). The anterior tibial translation (ATT), valgus tibial rotation (VTR), internal tibial rotation (ITR), Max.S on the TC, and ACL/graft forces were compared among the different simulation groups.

## 3 Results

### 3.1 Outcomes for intact knee, isolated ACL rupture, and ACL rupture with various additional injuries or knee defects

**FIGURE 3:**
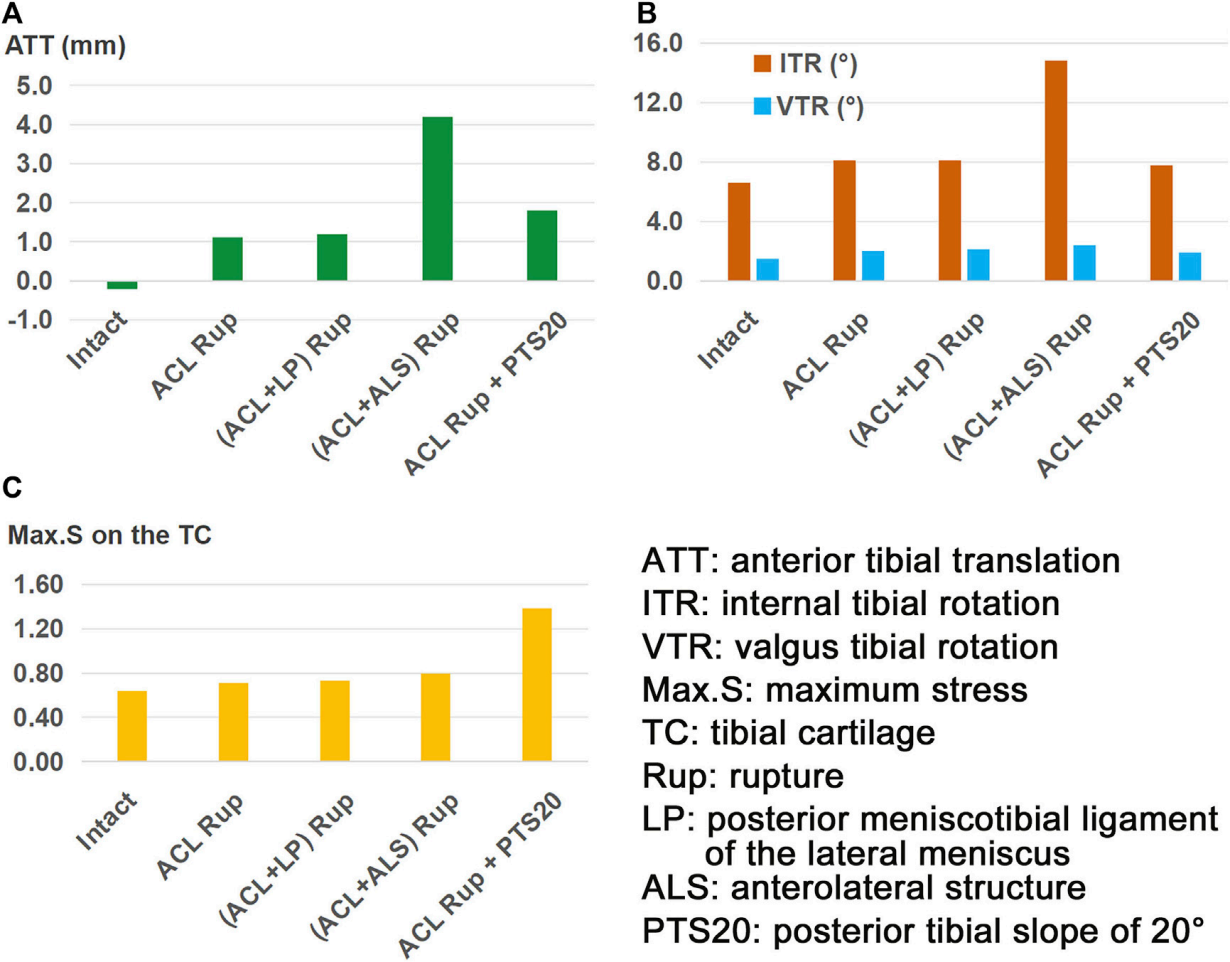
**(A)** Anterior tibial translation (ATT), **(B)** valgus tibial rotation (VTR) and internal tibial rotation (ITR), and **(C)** maximum von Mises stress (Max.S) on the tibial articular cartilage (TC) in response to a pivot shift loading condition in a knee with ACL rupture and different combinations of knee injuries or defects.

The knee displacements and articular stresses in the models at different knee states are shown in Figure 3. Compared to the intact state, the knee displacements and articular stresses increased after ACL rupture (−0.2 mm vs. 1.1 mm for ATT (Figure 3A), 6.7° vs. 8.1° for ITR, 1.5° vs. 2.0° for VTR (Figure 3B), 0.65 MPa vs. 0.71 MPa for Max.S on the TC). Compared with the isolated ACL rupture, all other injury models showed greater knee displacements and articular stresses. Damage to the ALS destabilized the knee more than a combined PTS of 20° or LP rupture (4.2 mm vs. 1.8 mm vs. 1.2 mm for ATT, 14.8° vs. 7.8° vs. 8.1° for ITR, and 2.4° vs. 1.9° vs. 2.1° for VTR, respectively). The knee with an ACL + LP rupture produced the most stable joint out of all combined injury models. In terms of the articular stress (Figure 3C), a PTS of 20° caused the highest Max.S on the TC, reaching over twice that of the intact knee (1.38 MPa vs. 0.64 MPa). The Max.S on the TC was higher after the ACL + ALS rupture than after ACL + LP rupture (0.80 MPa vs. 0.73 MPa).

### 3.2 Outcomes for ACLR with different combinations of knee injuries/defects

**FIGURE 4:**
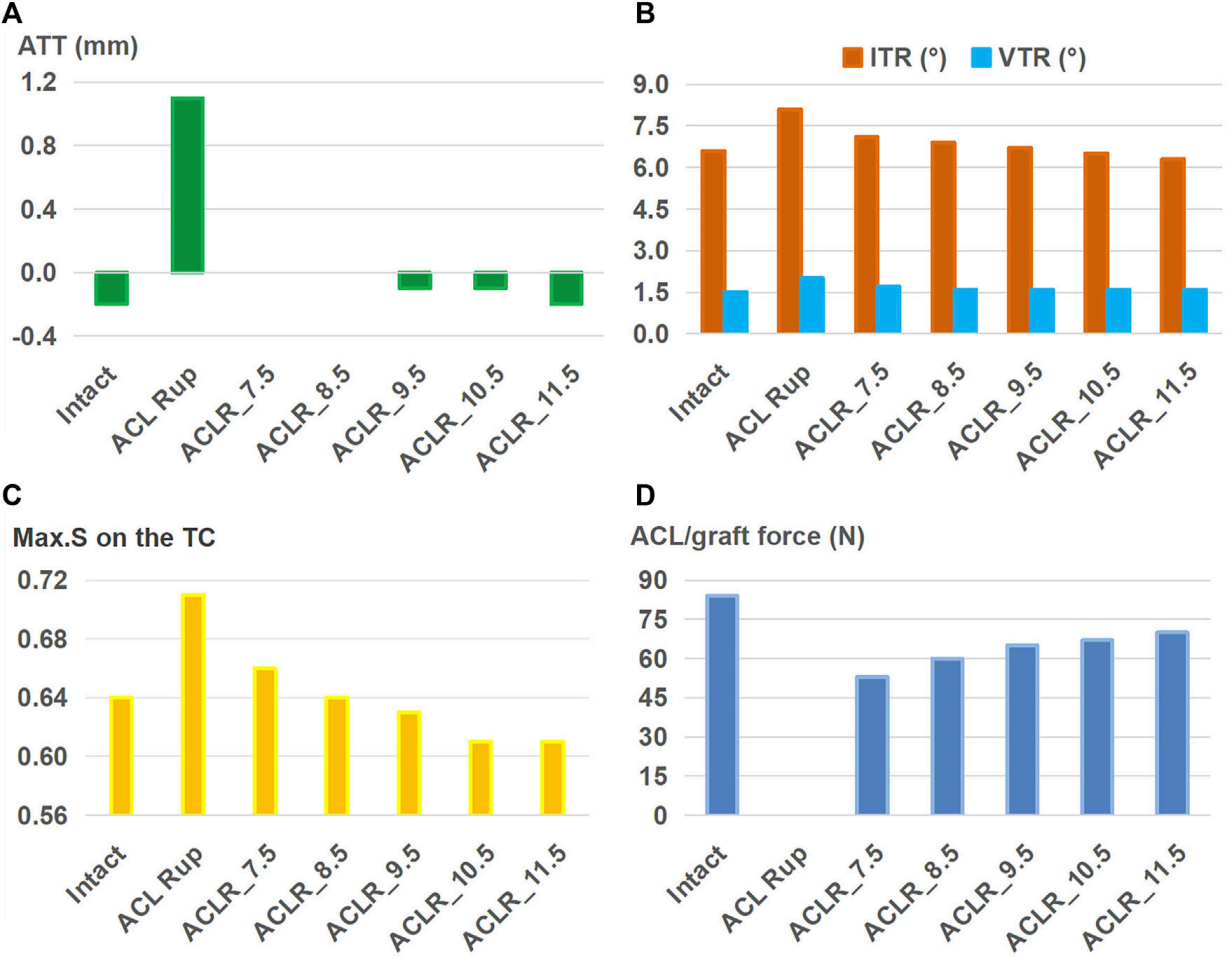
**(A)** Anterior tibial translation (ATT), **(B)** valgus tibial rotation (VTR) and internal tibial rotation (ITR), **(C)** maximum von Mises stress (Max.S) on the tibial articular cartilage (TC), and **(D)** graft forces in response to a pivot shift loading condition in a knee following ACL reconstructions (ACLRs) with varying graft diameters for treating isolated ACL rupture. Rup: rupture; ACLR_7.5: ACLR with a graft diameter of 7.5 mm.

The outcomes of the ACLR model with varying graft diameters (7.5–11.5 mm) for treating an isolated ACL rupture are shown in Figure 4. Compared to the ACL rupture model (ACL Rup), ACLR reduced ATT (Figure 4A), ITR, VTR (Figure 4B), and Max.S on the TC (Figure 4C) to restore the ligament force (Figure 4D) closer to the intact state. Compared to the intact group, a 7.5-mm-diameter ACL graft produced larger anterior (ATT) and rotational (ITR, VTR) tibial displacements (0 mm vs. −0.2 mm, 7.1° vs. 6.6°, 1.7° vs. 1.5°) as well as resulted in higher stresses on the tibial articular cartilages (0.66 MPa vs. 0.64 MPa for LTC, 0.38 MPa vs. 0.34 MPa), but the force in the graft was lower (53 N vs. 84 N). Each successive increase in graft diameter resulted in values closer to those of the intact model. The ATT and VTR were restored to the intact levels with a graft of diameter 11.5 mm, but the ITR and Max.S on the TC were restored close to the intact levels with 10.5 mm and 8.5 mm diameter grafts, respectively. Although increasing the graft diameter resulted in graft forces closer to the intact levels, there were noticeable differences even when using the largest graft. Specifically, the force was 70 N for ACLR with a graft of diameter 11.5 mm compared to 84 N in the intact state. Overall, ACLR with a graft of diameter 11.5 mm could restore the anterior and rotational knee stabilities as well as Max.S on the TC to within the intact level after isolated ACL rupture. However, the graft force was lower than that in the intact ACL.

**FIGURE 5:**
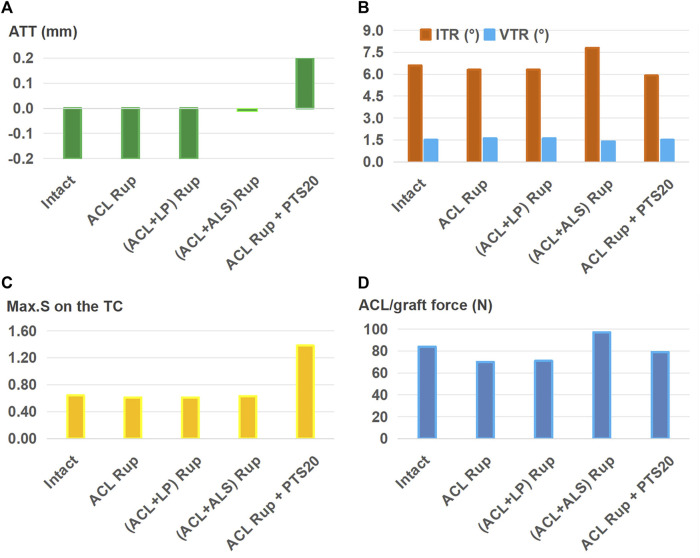
**(A)** Anterior tibial translation (ATT), **(B)** valgus tibial rotation (VTR) and internal tibial rotation (ITR), **(C)** maximum von Mises stress (Max.S) on the tibial articular cartilage (TC), and **(D)** graft forces in response to a pivot shift loading condition in a knee after ACL reconstruction (ACLR) with a graft of diameter 11.5 mm for treating different combinations of knee injuries. Rup, rupture; LP, posterior meniscotibial ligament of the lateral meniscus; ALS, anterolateral structure; PTS20, posterior tibial slope of 20°.

Based on the 11.5-mm graft, Figure 5 shows the results for ACLR after treatment with different combinations of knee injuries; it is seen that ACLR restored the anterior (Figure 5A) and rotational (Figure 5B) stabilities of the knee and articular stresses (Figure 5C) close to the intact levels when treating isolated ACL and ACL + LP ruptures. The graft forces (Figure 5D) in the groups simulating ACL and ACL + LP ruptures were also lower than that of the intact ACL after treatment by ACLR. The knee with an injury to the ALS still had greater anterior and internal rotational laxity as well as abnormally high graft force after treatment by ACLR when compared with the intact knee (0 mm vs. −0.2 mm for ATT, 7.8° vs. 6.6° for ITR, 97 N vs. 84 N for graft force). The knee with a PTS of 20° had a larger ATT (0.2 mm vs. −0.2 mm), and the Max.S on the TC was greater than that for the intact case (1.38 vs. 0.64 MPa).

### 3.3 Outcomes for ALSR with different combinations of knee injuries/defects

**FIGURE 6:**
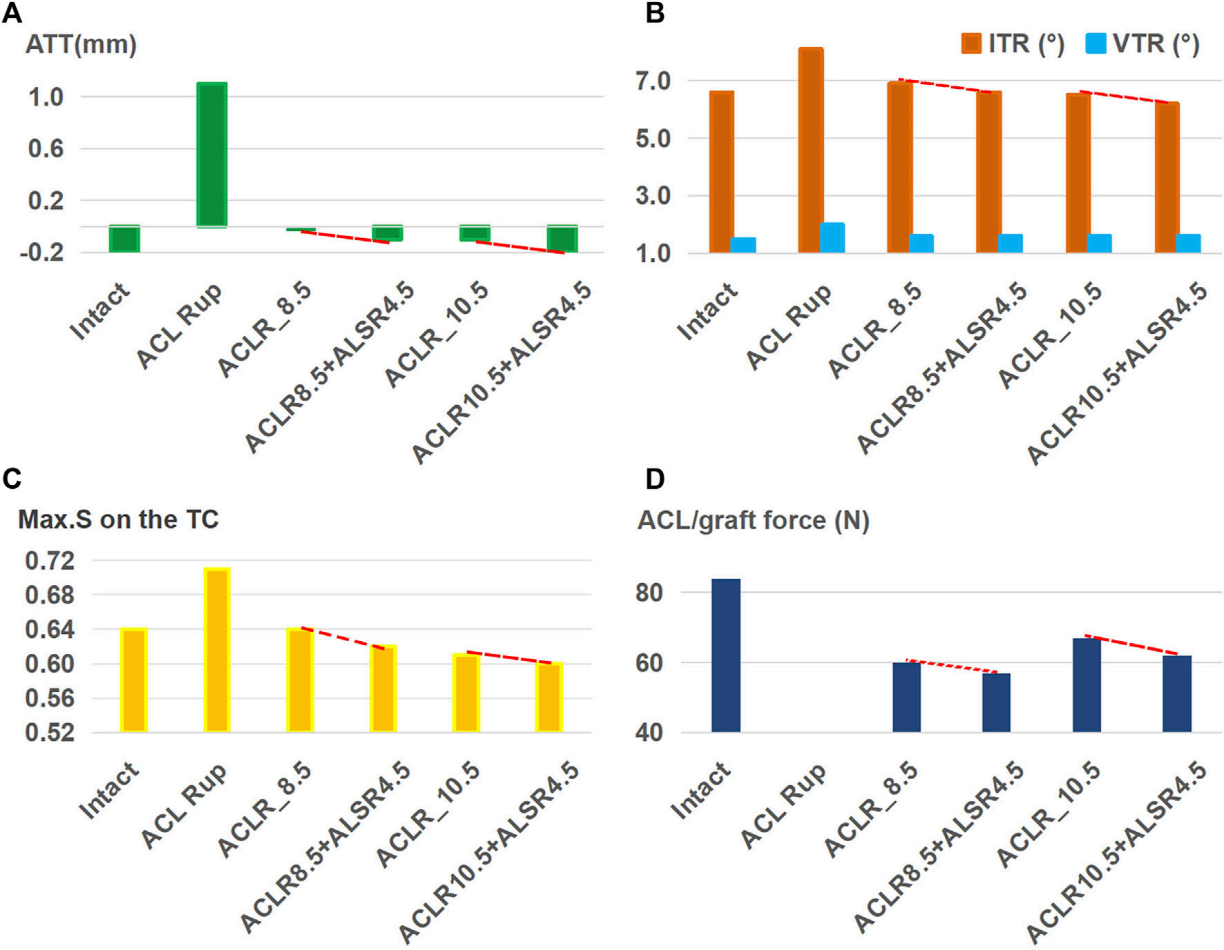
**(A)** Anterior tibial translation (ATT), **(B)** valgus tibial rotation (VTR) and internal tibial rotation (ITR), **(C)** maximum von Mises stress (Max.S) on the tibial articular cartilage (TC), and **(D)** graft forces in response to a pivot shift loading condition in a knee following I) ACL reconstructions (ACLRs) with graft diameters of 8.5 mm and 10.5 mm; II) anterolateral structures reconstruction (ALSR) with a graft of diameter 4.5 mm combined with ACLR with graft diameters of 8.5 mm and 10.5 mm. Both reconstruction techniques were used to treat an isolated ACL rupture. Rup, rupture; ACLR_8.5, ACLR with a graft of diameter 8.5 mm.

Figure 6 shows that when treating an isolated ACL rupture, ALSR (graft diameter: 4.5 mm) combined with ACLR (graft diameter: 8.5 mm) resulted in lower anterior (Figure 6A) and internal rotational (Figure 6B) displacements, articular stresses (Figure 6C), and graft forces (Figure 6D) than ACLR alone (−0.1 mm vs. 0 mm for ATT, 6.6° vs. 6.9° for ITR, 0.62 MPa vs. 0.64 MPa for Max.S on the TC, 57 N vs. 60 N for graft force). This trend was consistent between the two groups when using 8.5-mm and 10.5-mm diameter ACL grafts.

**FIGURE 7:**
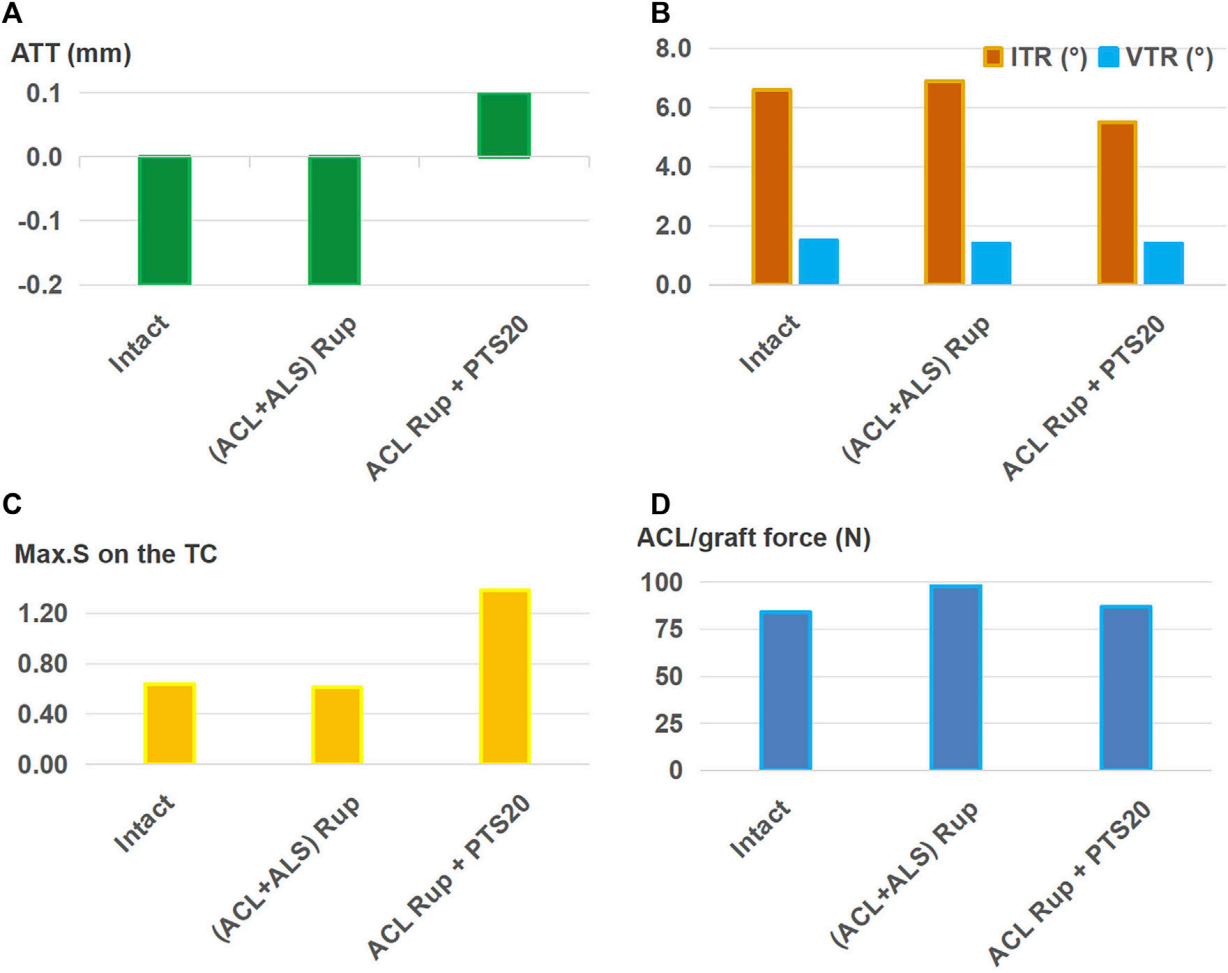
**(A)** Anterior tibial translation (ATT), **(B)** valgus tibial rotation (VTR) and internal tibial rotation (ITR), **(C)** maximum von Mises stress (Max.S) on the tibial articular cartilage (TC), and **(D)** graft forces in response to a pivot shift loading condition in a knee following anterolateral structures reconstruction (ALSR) (graft diameter: 4.5 mm) combined with ACLR (graft diameter: 11.5 mm). The procedure was used to treat different combined knee injuries. Rup, rupture; ALS, anterolateral structure; PTS20, posterior tibial slope of 20°.

As shown in Figure 7, ALSR combined with ACLR restored the ATT (Figure 7A), VTR (Figure 7B), and Max.S on the TC (Figure 7C) to normal levels for ACL rupture treatment accompanied by injury to the ALS. However, the ITR was still greater (6.9° vs. 6.6°) and graft force was higher (98 N vs. 84 N) than those for the intact case (Figure 7B). For the ACL rupture with a PTS of 20°, the ATT and Max.S on the TC were still greater than those of the intact knee (0.1 mm vs. −0.2 mm for ATT, 1.38 MPa vs. 0.64 MPa), and the graft force was higher than that of the intact ACL (87 N vs. 84 N, Figure 7D).

**FIGURE 8:**
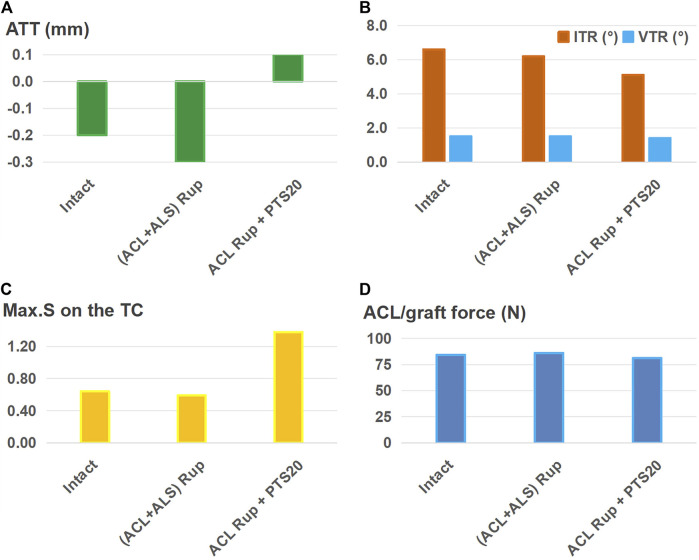
**(A)** Anterior tibial translation (ATT), **(B)** valgus tibial rotation (VTR) and internal tibial rotation (ITR), **(C)** maximum von Mises stress (Max.S) on the tibial articular cartilage (TC), and **(D)** graft forces in response to a pivot shift loading condition in a knee following anterolateral structures reconstruction (ALSR) (using a larger graft diameter of 6 mm) combined with ACLR (graft diameter: 11.5 mm). The procedure was used to treat different combined knee injuries. Rup, rupture; ALS, anterolateral structure; PTS20, posterior tibial slope of 20°.

Figure 8 shows that after using a larger graft diameter (6 mm) for ALSR, the knee displacement (Figures 8A, B), Max.S on the TC (Figure 8C), and graft force (Figure 8D) after ACL rupture accompanied by injury to the ALS were all restored to within the intact levels. However, for the ACL rupture with a PTS of 20°, the ATT and Max.S on the TC were still greater than those of the intact knee (0.1 mm vs. −0.2 mm for ATT, 1.38 MPa vs. 0.64 MPa for Max.S).

## 4 Discussion

It was found that compared to the isolated ACL rupture, combined injury to the LP, ALS, or increased PTS caused greater anterior and rotational knee laxity and articular stresses. Injury to the ALS caused the greatest knee laxity among all simulated conditions, and incorporating a PTS of 20° produced the largest Max.S on the TC. The joint stability and articular stress in the knee with isolated ACL rupture or ACL + LP rupture were restored close to those of the intact knee after treatment by isolated ACLR. An additional ALSR produced a lower pivot shift, articular stress, and force in the ACL graft than isolated ACLR, allowing better treatment of the ACL rupture with combined injuries to the ALS. ACLR combined with ALSR using a larger diameter of the ALSR graft (6 mm) than that suggested by the 2017 expert group consensus was shown to restore normal knee stability, articular stress, and graft force after injury to the ALS. However, even after treatment by ACLR combined with ALSR (graft diameter: 6 mm), incorporating a PTS of 20° resulted in greater ATT and Max.S on the TC than those for the intact knee. These findings may provide a scientific basis to further determine the surgical indications for ACLR combined with ALSR.

Compared to the intact state, both knee displacement and articular stress increased after isolated ACL rupture (Figure 3), indicating that the ACL is important for maintaining knee stability and normal articular stress. This is consistent with the findings of biomechanical and clinical follow-up studies that reported greater knee laxity and higher articular stress along with a high rate of long-term knee osteoarthritis after ACL injury (Arokoski et al., 2000; Barenius et al., 2014; Wang et al., 2020b). Injury to the ALS caused a larger knee displacement and maximum stress on the TC than injury to the LP likely because of the lateral location of the ALS on the knee joint, which plays a more important role in restraining the movement of the femoral condyle toward the tibial plateau than the more centrally located LP. The results of this study also show that compared with isolated ACL rupture, the ATT increases when combined with increased PTS, which is consistent with the findings of a previous study (Bauer et al., 2022). However, the ITR reduced slightly with the higher PTS, which could be caused by lifting of the anterior region of the tibial plateau as it rotates posteriorly. This might restrain the joint in the anterior space and produce less tibial rotation. Similarly, a PTS of 20° resulted in higher Max.S on the TC than injury to the LP or ALS, which could be attributed to the restrained motion from lifting of the anterior tibial plateau. In this study, the lowest values of knee laxity and articular stress were obtained after ACL rupture with an associated injury to the LP (Figure 3). Similarly, Tang et al. (2019) reported that the ATT in a knee placed under a simulated pivot-shift load increased only slightly after tearing of the posterior meniscal root.

ACLR was shown to reduce knee laxity and lower the Max.S on the tibial articular cartilage after isolated ACL rupture; it was also more effective as the diameter of the graft increased (Figure 4), which was consistent with literature (Wang et al., 2020b). Knee stability and articular stress were completely restored to normal levels when using a relatively large graft diameter (11.5 mm) to treat isolated ACL and ACL + LP ruptures (Figure 5). However, a large graft diameter may cause graft impingement on the femoral notch and increase the risk of early graft rupture after surgery (Dayan et al., 2015). Considering the large individual variations in the Young’s modulus of the ACL grafts reported in previous studies (5–1500 MPa) (Smeets et al., 2017; Jacquet et al., 2020), grafts with larger values than those used in the present study may require smaller graft diameters for restoring the knee stability and articular stress to normal levels (Wang et al., 2022b).

This study also showed that ALSR further reduced the anterior and rotational knee displacements, articular stress, and ACL graft force compared to those of isolated ACLR (Figure 6). These outcomes are consistent with reports by Nitri et al. (2016) and Ueki et al. (2019). It is also postulated that ALSR may allow the use of a smaller graft diameter in ACLR and hence lower the risk of graft impingement on the femoral notch. This study identifies that injury to the ALS cannot be satisfactorily treated with the isolated ACLR or combination of ACLR and ALSR when using a graft of diameter 4.5 mm for the ALSR (Figure 7); however, successful treatment may be achieved with a combination of ACLR and ALSR when using a larger graft diameter (6 mm) for the ALSR (Figure 8). Moreover, a PTS of 20° still resulted in larger ATT and Max.S on the TC than those of the intact knee, even after treatment by a combination of ALSR (graft diameter: 6 mm) and ACLR (graft diameter: 11.5 mm). In this condition, an osteotomy may be needed to achieve better knee functionality and stress distribution on the articular cartilage.

The present study has some notable limitations. 1) The surgical technique for ALSR was according to the recommendations of the 2017 expert consensus (Sonnery-Cottet et al., 2017). Different surgical techniques, such as varying tunnel positions, were not explored in this study, which could affect the outcomes of ALSR. However, these factors were maintained constant across different simulation groups to exclude their impacts on the comparison outcomes. 2) The finite element model was adopted from a single subject, which may not represent the conditions of other subjects. Additionally, the Young’s modulus of the ACL graft was obtained from literature, but it could vary with individual differences. However, the single finite element model allows the variate to be controlled to one factor, which can be modified infinitely without causing damage to the sample. This permits the basic biomechanics to be revealed more clearly. Future research efforts could therefore include both cadaveric and *in vivo* studies to further validate the findings of this study. *In vivo* studies are particularly advantageous for examining the effects of individual differences and incorporating the effects of joint muscles. 3) Only a static loading condition was considered in this study, and complex loading environments representing different daily activities were not simulated. 4) Viscoelasticity and initial tension values of the ligaments were not considered in this study. In reality, stress relaxation occurs when the joint is subjected to external loading, and the initial tension of the ligaments can enhance their ability to better resist these loads. Therefore, the translations and rotations of the knee joint calculated in this study may have some systematic errors.

## 5 Conclusion

This study showed that combined injury to the LP and ALS with higher PTS angles led to higher degrees of anterior and rotational knee laxity and higher articular stresses in the ACL-deficient knee models. Injury to the ALS caused the greatest knee laxity among all conditions simulated, and incorporating a PTS of 20° produced the largest Max.S on the TC. Correspondingly, these combined injuries need to be treated with different surgical strategies to prevent postoperative pivot shifts and restore the articular stresses and graft forces to those similar to the intact knee. Using a human cadaver knee model, this study showed completely restored knee stability and articular stress after isolated ACL rupture or ACL rupture with combined LP injury when treated by isolated ACLR. Knee biomechanics after combined rupture of the ACL and ALS were only restored to those similar to the intact knee when treated by ACLR combined with ALSR using a large ALSR graft diameter (6 mm). However, ACL rupture combined with a PTS of 20° may need an additional strategy, such as osteotomy, to prevent pivot shift and reduce the risk of articular cartilage degeneration after ACLR.

## Data Availability

The original contributions presented in the study are included in the article/Supplementary Material, and any further inquiries may be directed to the corresponding authors.

## Abbreviations

ACL, anterior cruciate ligament; ACLR, anterior cruciate ligament reconstruction; ALS, anterolateral structure; ALL, anterolateral ligament; ALC, anterolateral capsule; LP, posterior meniscotibial ligament of the lateral meniscus; PTS, posterior tibial slope; ALSR, anterolateral structures reconstruction; Max.S, maximum von Mises stress; TC tibial cartilage; ATT, anterior tibial translation; VTR, valgus tibial rotation; ITR, internal tibial rotation; Rup, rupture.
